# An Interesting Case of Weakness and Atrial Tachycardia in the Emergency Department: Thinking Beyond Hearts and Minds

**DOI:** 10.7759/cureus.38002

**Published:** 2023-04-23

**Authors:** Ali Mohammed B Yahya, Nasser Ahmed, Hasan Qayyum

**Affiliations:** 1 Emergency Department, Sheikh Khalifa Medical City, Abu Dhabi, ARE

**Keywords:** emergency medicine resuscitation, electrocardiography (ecg), reversible cause of muscle weakness, thyrotoxic hypokalemic periodic paralysis, other causes of hypokalemia

## Abstract

Thyrotoxic periodic paralysis is a rare but life-threatening presentation of hyperthyroidism that manifests with sudden, painless episodes of muscle weakness due to hypokalemia. We present the case of a middle-aged Middle Eastern female who attended our Emergency Department with sudden onset weakness to the lower limbs, resulting in her inability to walk. She had a power of 1/5 in the lower limbs, and subsequent investigations showed a low potassium level, and primary hyperthyroidism secondary to Grave's disease was diagnosed. A 12-lead electrocardiogram showed atrial flutter with a variable block, along with U waves. The patient reverted to sinus rhythm following administration of potassium replacement and was also treated with Propanalol and Carbimazole. The patient made a full neurological recovery.

Emergency physicians and all frontline healthcare workers should be aware that electrolyte problems can cause paralysis. Furthermore, hypokalemic periodic paralysis can be caused by an undiagnosed thyrotoxic state. Be aware that if left untreated, hypokalemia can cause serious atrial and ventricular arrhythmias. Achieving a euthyroid state and blunting hyperadrenergic stimulation, in addition to replacing potassium, all help to fully reverse muscle weakness.

## Introduction

Hyperthyroid hypokalemic periodic paralysis (HHPP) is a rare but life-threatening presentation of hyperthyroidism that manifests with sudden, painless episodes of muscle weakness due to hypokalemia. It is more common in the Asian population and is often provoked by heavy exercise or a carbohydrate-rich meal [1]. Sometimes no specific trigger is found. HHPP can present with a variety of electrocardiographic changes, including atrial and ventricular tachyarrhythmias [2]. The management in the emergency department should focus on not just replacing potassium but also reducing the hyperadrenergic state with non-selective beta blockers. The neurological deficit, once treated, is completely and rapidly reversible [3].

## Case presentation

A 49-year-old female of Middle Eastern extraction presented to our emergency department (ED) with a history of weakness in both lower limbs and generalized malaise. This weakness was sudden in onset, originating with a feeling of malaise two days prior. It was described as weakness and fatigue in all four limbs but was predominantly in the lower limbs, resulting in her inability to walk on the day of ED attendance. Pertinent negative history included no associated fever, headache, blurred vision, facial drooping, sphincter disturbances, or abdominal pain. There was also no history of trauma, alcohol ingestion, or substance misuse. She had no significant past medical history of note and was not on any regular medication. 

On examination, vital signs were recorded as within normal limits in an awake and alert patient, except for a resting tachycardia of 106 beats per minute. The patient was afebrile. A neurological examination revealed no cranial nerve palsies. Power in the upper limbs was recorded as 4/5 and in the lower limbs as 1/5. Deep tendon reflexes were all reduced. Sensation was intact in all four limbs. The capillary blood glucose was within normal limits.

**Figure 1:**
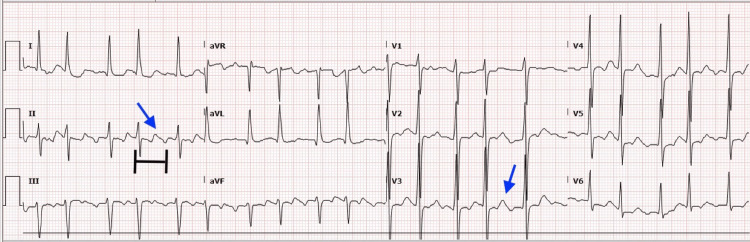
12-lead electrocardiograph with a potassium level of 2.4 mmol/l An irregular, narrow, complex rhythm at a rate of 126 beats per minute with a saw-tooth baseline consistent with an atrial tachycardia, most likely an atrial flutter with a variable block, is demonstrated. U waves are also visible as a positive deflection after the T wave, as seen here in lead V3 and lead II (blue arrows). The corrected QT interval was 397 milliseconds. In hypokalemia with broad-based U waves, the QT interval is sometimes called the QU interval, as seen here with black callipers.

As part of the patient’s workup, a 12-lead electrocardiogram was recorded at first medical contact, as shown in Figure 1. This revealed an irregular, narrow, complex rhythm at a rate of 126 beats per minute. A saw-tooth baseline is consistent with atrial tachycardia, most likely, an atrial flutter with a variable block was demonstrated on the 12-lead electrocardiogram. U waves were also visible as a positive deflection after the T wave, as seen here in lead V3 and lead II. The corrected QT interval was 397 milliseconds. In hypokalemia with U waves, the QT interval is sometimes called the QU interval [4].

Initial laboratory results showed a serum potassium level of 2.4 mmol/l (reference range: 3.4 to 5.1 mmol/l). Also recorded was a low phosphate level of 0.61 mmol/l (reference range: 0.81 to 1.45 mmol/l). The patient was started on a potassium replacement infusion of potassium chloride at a rate of 10 mmol per hour. 

**Figure 2:**
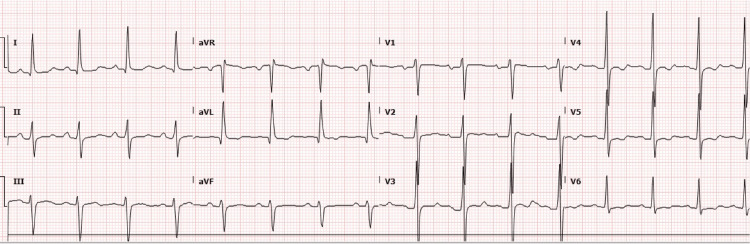
Repeat 12-lead electrocardiograph showing normal sinus rhythm The patient reverted to sinus rhythm approximately one hour after the commencement of potassium replacement. Repeat potassium on the same day was corrected to 4.1 mmol/l.

As shown in Figure 2, the patient reverted to a normal sinus rhythm at 94 beats per minute approximately one hour after potassium replacement was commenced. The subsequent potassium concentration, when rechecked the same day, was now corrected at 4.1 mmol/l. 

Further, workup in ED showed an undetectable thyroid stimulating hormone level at 0.005 milli IU/L (reference range 0.27 to 4.2 milli IU/L) and high free thyroxine levels at 98.7 pmol/L (reference range 12 to 22 pmol/L) and high free tri-iodothyronine levels at 30.4 pmol/L (reference range 3.1 to 6.8 pmol/L), consistent with thyrotoxicosis. Thyrotropin receptor antibodies were positive at 26 IU/L. Laboratory results are displayed in Table 1.

**Table 1:** Laboratory results Laboratory results in the emergency department showed severe hypokalemia, hypophosphatemia, and thyroid function tests consistent with thyrotoxicosis.

Laboratory Results (units)	Level	Reference Range
Sodium (mmol/L)	141	136-145
Potassium (mmol/L)	2.4	3.2-5.5
Magnesium (mmol/L)	0.77	0.66 – 1.07
Glucose (mmol/L)	7.8	3.9-6.0
Calcium (mmol/L)	2.41	2.15-2.55
Phosphorus (mmol/L)	0.61	0.81-1.45
Creatine Kinase (IU/L)	65	39-308
Thyroid Stimulating Hormone (milli IU/L)	0.005	0.27-4.20
Free Thyroxine (pmol/L)	98.7	12-22
Free Tri-iodothyronine (pmol/L)	30.4	3.1-6.8

After endocrine consultation, the patient was commenced on propranolol and carbimazole and was given a final diagnosis of thyrotoxic hypokalemic periodic paralysis secondary to Graves’ disease.

The patient made a full neurological recovery within four hours of potassium replacement. She was subsequently discharged 48 hours later after internal medicine and endocrinology consultations. An outpatient ultrasound of the thyroid gland was arranged, but the patient was unable to follow up.

## Discussion

Hypokalemia is commonly diagnosed in the Emergency Department, often resulting from gastrointestinal losses and medication use (e.g., diuretics) [4]. Hypomagnesemia is often associated with hypokalemia.

Hypokalemic periodic paralysis is a type of primary periodic paralysis characterized by painless paroxysms of muscle weakness. The causes can be familial and are attributed to mutations in the Cav1.1 skeletal muscle calcium channel and the Nav1.4 Na+ channel. Non-familial causes include thyrotoxic periodic paralysis (TPP) and sporadic periodic paralysis (SPP), which are more common in Asians and Hispanics [3]. Thyrotoxic periodic paralysis has a male preponderance [5]. Other primary periodic paralysis types are hyperkalemic periodic paralysis and Anderson-Tawil syndrome [6].

Although the prevalence of hyperthyroid hypokalemic periodic paralysis in the UAE is difficult to estimate due to population migration [5], the incidence in Asian countries (92%) is approximately 10-20 times higher than non-Asian ethnic populations [3]. Cases have been sporadically described in the Arab population [6]. 

Thyrotoxic periodic paralysis has a male preponderance [7]. Other primary periodic paralysis types are hyperkalemic periodic paralysis and Anderson-Tawil syndrome [8].

Hypokalemic periodic paralysis is characterized by a triad of muscle paralysis, acute hypokalemia without total body potassium deficit, and hyperthyroidism [3].

The exact pathophysiology of HHPP is unclear but involves increased Na+-K+ ATPase activity, directly induced by thyroid hormone and indirectly by hyperadrenergic activity. In addition to this, reduced potassium efflux leads to the trapping of potassium in cells. As a result, a vicious cycle of hypokalemia-induced paradoxical depolarization and inactivation of sodium channels leads to muscle inexcitability and paralysis [3].

Certain susceptibility loci have been identified that influence the expression of the KCNJ2 gene. This gene encodes an inwardly rectifying potassium channel called Kir2.6, expressed in skeletal muscle and transcriptionally regulated by thyroid hormone. The frequency of expression of these genes varies across ethnicities, which may explain why HHPP is more common in some ethnicities than others [9-11]. 

Any event that increases insulin or epinephrine, resulting in an inward flux of potassium, can precipitate an episode of paralysis [12,13]. Common triggers are rest following heavy physical activity or a high carbohydrate load. Other precipitants include exposure to colds, concurrent infections, alcohol intake, steroid therapy, beta-agonist bronchodilator use, and menstruation. Sometimes no specific precipitating factor is identified [14,15].

Episodes of paralysis typically last a few hours but can range from minutes to a few days. The frequency of episodes also varies. Most episodes occur in the early hours of the day and are also reportedly more common in the summer, as was the case in our case [16,17]. The first episode of paralysis usually occurs between the ages of 5 and 35, but it is most common between the ages of 15 and 35 years [18,19].

HHPP typically presents with sudden-onset muscle weakness, with the lower limbs being first involved, followed by ascending paralysis [20]. The paralysis resolves in the reverse order in which it was initiated [2]. The absence of sensory loss and lack of cranial nerve palsies differentiate it from other neurological conditions like Guillain-Barré Syndrome and its variants.

Mild myalgia is present in only half of all cases [12]. Reduced or absent deep tendon reflexes are common. Sensation and conscious levels are unimpaired. Clinical features of thyrotoxicosis, like warm, moist skin, resting tachycardia, goiter, and exophthalmos, are often subtle but important to pick up [2].

Laboratory results universally show a low serum potassium level. However, this may often be mild, with levels less than 3.5 meq/L reported [8]. The degree of hypokalemia can correlate with the severity of weakness [21]. Low phosphate and magnesium levels are also reported, in addition to mildly elevated creatine kinase levels. Abnormal thyroid function tests suggestive of thyrotoxicosis are seen (low thyroid stimulating hormone; elevated free and total thyroxine and tri-iodothyronine; increased tri-iodothyronine uptake) [2]. In thyrotoxicosis due to Graves’ disease, elevated thyroid receptor antibodies are seen along with hypervascularity on thyroid ultrasound and increased tracer uptake on a radioactive iodine uptake thyroid gland scan [22]. 

Frontline workers should be aware of ECG changes in hypokalemia, a common diagnosis made in the emergency department. Classic changes described in the literature include a flattened or inverted T wave, often the earliest sign of hypokalemia. Other classical changes are ST segment depression, U waves, prolongation of the PR interval, prolongation of the QTc interval, premature beats, and tachyarrhythmias [23]. Recently, an apparent long QT interval has also been described from the fusion of T and U waves, known as a long QU interval [24].

U waves, a small positive deflection seen after the T wave, are caused by a prolongation of the recovery phase of the cardiac action potential [23]. They are almost always seen in hypokalemia but can also be found in patients taking medications that block potassium channels. 

Severe hypokalemia (serum potassium level less than 2.5 mmol/l), as in our case, can manifest with a multitude of tachyarrhythmias, including ventricular tachycardia, torsade de pointes, atrial flutter, atrial fibrillation, and rarely atrioventricular block [23,24].

A triad of electrocardiographic findings that is not always present but may allude to HHPP includes sinus tachycardia, a long QU interval, and a paradoxically prolonged PR interval due to thyrotoxicosis (paradoxical as sinus tachycardia is usually associated with a relatively short PR interval) [25]. Increased QRS amplitude has also been reported along with atrial and ventricular arrhythmias [2,25].

Preventative advice to avoid precipitating factors in HHPP, like excessive strenuous activity, high-carbohydrate meals, and alcohol consumption, has been suggested [1]. Pharmacological therapy focuses on treatment to abort acute attacks and chronic preventive strategies to reduce their frequency [8]. Treatment in the acute phase includes potassium replacement. This can be administered orally or intravenously, the latter being used in severe hypokalemia or where swallowing is impaired. It should be mentioned that there is a risk of rebound hyperkalemia in 40% to 80% of treated cases [16,26]. This occurs due to the release of potassium and phosphate from the cells on recovery [16,27]. In addition to potassium replacement, in the acute phase, a nonselective beta-blocker like propranolol will help limit Na+-K+-ATPase activity by blunting hyper-adrenergic stimulation, thereby preventing intracellular shifts of potassium and phosphate [28]. Administration of the non-selective beta-blocker in addition to methimazole (or another antithyroid agent) prevents relapse of paralysis and aids in returning to a normal functioning life until definitive treatment of hyperthyroidism occurs [29].

Definitive treatment involves achieving a euthyroid state, which can eliminate future paralytic episodes. This may include antithyroid medication, surgery, or radioiodine therapy [3,14,28,30,31]. 

Carbonic anhydrase inhibitors have no role in TPP and may increase the frequency of paralytic episodes [20].

## Conclusions

It is important to investigate the etiology of hypokalemia in patients presenting to the emergency department. Often the differentials extend beyond the apparent cause, as in our case, where thyrotoxicosis as a cause of hypokalemia was diagnosed.

Emergency physicians and all frontline healthcare workers should be aware that electrolyte problems can also cause paralysis. Furthermore, hypokalemic periodic paralysis can be caused by an undiagnosed thyrotoxic state. Be aware that if left untreated, hypokalemia can cause serious atrial and ventricular arrhythmias. Timely consultation with endocrinology and achieving a euthyroid state, in addition to replacing potassium, help to correct a weakness.
